# Supplementary material for: Abdominal imaging associates body composition with COVID-19 severity
Source: PLoS One. 2023 Apr 13;18(4):e0283506. doi: 10.1371/journal.pone.0283506 (PMC10101472; doi:10.1371/journal.pone.0283506)
Supplement: Attachment — Submitted filename: COVIDpaperresponse_2.pdf [file pone.0283506.s005.pdf]

1. Please ensure that your manuscript meets PLOS ONE's style requirements, including those for file naming. The PLOS ONE style templates can be found at

https://journals.plos.org/plosone/s/file?id=wjVg/PLOSOne_formatting_sample_main_body.pdf and

https://journals.plos.org/plosone/s/file?id=ba62/PLOSOne_formatting_sample_title_authors_affiliations.pdf

We have edited the manuscript accordingly and made changes to match the house style including size and bold font section heading, numbered equations, replaced Figure with Fig in the text, moved table titles to the top of the tables, renamed supplementary tables to S1 Table throughout text, and moved the supporting information to the end of the manuscript after the reference list.

2. Please provide additional details regarding participant consent. In the ethics statement in the Methods and online submission information, please ensure that you have specified what type you obtained (for instance, written or verbal, and if verbal, how it was documented and witnessed). If your study included minors, state whether you obtained consent from parents or guardians. If the need for consent was waived by the ethics committee, please include this information.

We have clarified the text detailing the ethics and consent covering this study in the study design section.

3. Thank you for stating the following in the Acknowledgments Section of your manuscript:

"This work was made possible by the UK Biobank, including staff, funders, and study volunteers. We thank Alan Young and Howard Callen at UK Biobank for facilitating data access, and Amoolya Singh, Chang-Heok Soh and Neha Murad for helpful feedback on the manuscript. This research has been conducted using the UK Biobank Resource under Application Number 44584 and was funded by Calico Life Sciences LLC."

We note that you have provided additional information within the Acknowledgements Section that is not currently declared in your Funding Statement. Please note that funding information should not appear in the Acknowledgments section or other areas of your manuscript. We will only publish funding information present in the Funding Statement section of the online submission form.

Please remove any funding-related text from the manuscript and let us know how you would like to update your Funding Statement. Currently, your Funding Statement reads as follows:

"This research was funded by Calico Life Sciences LLC.

https://www.calicolabs.com"

Please include your amended statements within your cover letter; we will change the online submission form on your behalf.

We have removed reference to funding from the acknowledgement. The corrected funding statement is:

This research was funded by Calico Life Sciences LLC. https://calicolabs.com

In addition to that, for the latest revision request, here is an updated statement:

Calico Life Sciences LLC provided support in the form of salaries for authors EPS, MC, and RS, but did not have any additional role in the study design, data collection and analysis, decision to publish, or preparation of the manuscript. JDB and ELT hold a Research Grant from Calico LLC, which funds BW, NB and MT's employment at the University of Westminster.

4. In your Data Availability statement, you have not specified where the minimal data set underlying the results described in your manuscript can be found. PLOS defines a study's minimal data set as the underlying data used to reach the conclusions drawn in the manuscript and any additional data required to replicate the reported study findings in their entirety. All PLOS journals require that the minimal data set be made fully available. For more information about our data policy, please see http://journals.plos.org/plosone/s/data-availability.

Upon re-submitting your revised manuscript, please upload your study's minimal underlying data set as either Supporting Information files or to a stable, public repository and include the relevant URLs, DOIs, or accession numbers within your revised cover letter. For a list of acceptable repositories, please see http://journals.plos.org/plosone/s/data-availability#loc-recommended-repositories. Any potentially identifying patient information must be fully anonymized.

Important: If there are ethical or legal restrictions to sharing your data publicly, please explain these restrictions in detail. Please see our guidelines for more information on what we consider unacceptable restrictions to publicly sharing data: http://journals.plos.org/plosone/s/data-availability#loc-unacceptable-data-access-restrictions. Note that it is not acceptable for the authors to be the sole named individuals responsible for ensuring data access.

We will update your Data Availability statement to reflect the information you provide in your cover letter.

Our research was conducted using UK Biobank data. Under the standard UK Biobank data sharing agreement, researchers are unable to directly share data they obtain from the biobank with others. However, all of the the the data generated and used in this paper are available directly from the UK Biobank upon request at https://www.ukbiobank.ac.uk and we have included this in the data availability statement.

Additional Editor Comments (if provided):

The paper has been evaluated by two expert reviewers. Both reviewers recognize the strengths of the study but they have also identified a number of major and minor issues that need to be addressed before the manuscript can be considered for publication. In addition to the points raised by the Reviewers, I would ask the authors to more clearly describe 1) in subsection "Predictive modeling" whether the data was split on the subject level in the cross-validation and 2) Throughout the manuscript what data exactly was used as input in these models (differences between baseline and follow-up vs both time points; this is not very clear everywhere).

We would like to thank the editor for this comment, we have addressed this point in the methods and the paper has clearly benefited from this added clarity. We have split the methods into subsections, clearly differentiating the longitudinal analysis and the baseline-only analyses. The predictive modeling was performed on the

baseline data only, as we tried to predict disease severity from image-derived body composition before the pandemic. The data were not paired at subject level in the predictive analysis as only one time point was used, and therefore could not be split.

Reviewer #1: This study performed a large-scale analysis for abdominal imaging associates body composition with COVID-19 severity. This is timely needed study with comprehensive analysis and decent writing.

Does the author perform a human involved QA process for image processing results. I can imagine some segmentation results are failed.

This is a good point to raise, quality control with big data has to be very stringent, and we did indeed perform extensive QA by visually checking the fifty largest and fifty smallest volumes, as well as another 50 random for each structure. The QA protocol is outlined in [37], however we have added some additional details into the manuscript to explain this more clearly.

It would be helpful to illustrate the rationale of using image processing pipeline [37].

We thank the reviewer for pointing this out. The preprocessing steps, segmentation models for individual organs and the quantitative analysis of PDFF (proton density fat fraction) and R2* have been applied to approximately 38,000 scans from the UK Biobank in Liu et al. (2021). We have added text in the "Image-derived phenotypes" section that provides the rationale.

Why so many factors have large confidence intervals in Figure 2 and 3?

The variables affected by large confidence intervals in Figure 3 (smoking and alcohol) are self-reported, and therefore have more variability compared to quantitative values; there could also be a bias in answering those questions, given their negative connotation. For Figure 3, the confidence intervals are also quite large for sex as well as ethnicity and imaging centre. We believe this is due to the uneven distribution of severe cases as for example there are about three times more severe cases in men in that particular dataset.

It seems the trend of results is not consistent between Figure 2 and 3 for statistical significance.

We thank the reviewer for this remark, the plots are quite similar, but the outcome variable is different. Figure 2 summarises the changes between the two timepoints between cases and controls, whereas Figure 3 is about investigating the associations between image-derived phenotypes (IDPs) and severity. We have made this more explicit in the text and captions.

From Figure 4a, the BMI is a much more impactful feature compared with Age. But in Figure 4b, their importance are almost the same. That is somehow contradictory.

Thank you for raising this point.. Figure 4A are the ROC curves for the three different models (Age+Sex, Age+Sex+Height+BMI, Age+Sex+BMI+Height+IDPs)

whereas figure 4B is the importance of the random forest features for the last model only. We have changed the caption to reflect this.

Why the visceral fat and liver fat are not used in the Figure 4a?

Thank you for this question, which falls into the same category as the one above. We did use visceral fat and liver fat in figure 4A, the green line represents the model with Age+Sex+BMI+IDPs, where IDPs are the image-derived phenotypes (visceral fat and liver fat, iliopsoas muscle volume). We have amended the figure caption accordingly to remove any ambiguity.

It seems the top predictors are all fat related features, which would have strong interaction.

Yes, we agree that several of the key variables are likely to correlate with each other. We included a regularisation term in the regression model, using the LASSO (Least Absolute Shrinkage and Selection Operator), to perform variable selection and produce a more parsimonious and interpretable result.

Reviewer #2: In this study the authors analyzed data of UK Biobank recruited recovered SARS-CoV-2 positive individuals (n=967) and matched controls (n=913) who were extensively imaged prior to the pandemic and underwent follow-up scanning. They specifically investigated longitudinal changes in body composition, as well as the associations of pre-pandemic image-derived phenotypes with COVID-19 severity. The authors found a decrease in lung volume with SARS-CoV-2 positivity. They also observed that increased visceral adipose tissue and liver fat, and reduced muscle volume, prior to COVID-19, were associated with COVID-19 disease severity. In addition, when training a machine classifier with demographic, anthropometric and imaging traits, they found that visceral fat, liver fat and muscle volume have prognostic value for COVID-19 disease severity beyond the standard demographic and anthropometric measurements. The authors have much expertise in this field of research and the topic addressed by this study is important.

Specific comments:

1. Introduction: When the authors address post-acute sequelae of COVID-19 they should also discuss the findings of very important studies that investigated this question (Nature. 2021 Jun;594(7862):259-264; Nat Med. 2022 Mar;28(3):583-590; Lancet Diabetes Endocrinol. 2022 Mar 21:S2213-8587(22)00044-4. doi: 10.1016/S2213-8587(22)00044-4).

We thank the reviewer for highlighting these papers. Given the extraordinary number of relevant papers that have been published since the pandemic began it was not possible to include all of the references we would have liked. At the time of writing we focussed on the most organ specific publications most directly relevant to this manuscript. We however have expanded our Introduction to include the suggested texts and the paper clearly benefits from this addition.

2. Introduction: The authors should also discuss how the COVID-19 pandemic may generally impact on the cardiometabolic risk and on obesity (Nat Rev Endocrinol. 2021 Mar;17(3):135-149).

We have expanded the part of the discussion where we describe how chronic comorbidities such as obesity, impaired metabolic health, and diabetes are known to be at higher risk of severe disease and poorer prognosis following COVID-19 infection to include this excellent review by Stefan et al.

3. Impaired cardiometabolic health and obesity are thought to promote SARS-CoV-2 vaccine-breakthrough infections (Nat Rev Endocrinol. 2022 Feb;18(2):75-76). Do the authors have data to address this important point? Otherwise, they should at least carefully discuss this point.

The referee raises an important point. Unfortunately we do not have any specific data available to us within this cohort regarding vaccine-breakthrough infections however we have included a reference to this in the introduction.

4. When the authors address the relationship of obesity with severity of COVID-19, they should also discuss the important findings from the QResearch database of general practices in England, UK study (Lancet Diabetes Endocrinol. 2021 Jun;9(6):350-359), which carefully investigated the relationship of obesity, under the aspects of different comorbidities and age, with the severity of COVID-19.

We have added this reference to the text.

5. On what basis were specifically the 1,880 participants, who attended the first imaging visit, selected and recruited to the new re-imaging study? It appears as if these 1,880 participants are the cases and controls. So, what was the initial number of subjects with a positive SARS-CoV-2 test result that was asked to attended the second imaging visit?

The UK Biobank rescanning of participants for the COVID study initially aimed to recruit 2,000 participants, the inclusion criteria are listed in detail here: https://biobank.ndph.ox.ac.uk/showcase/showcase/docs/casecontrol_covidimaging.pdf, but in brief participants needed to: have already attended pre-pandemia imaging assessment at one of the three imaging sites, live within 60km of the clinic (COVID travel restrictions were still in place during the rescanning study) and had high-quality UK Biobank MRI images available from their first imaging visit. These criteria are in our section “study design” on page 6. At the time of preparing this manuscript, a total of 1,880 suitable datasets were available for the study.

6. The authors assessed longitudinal effects of SARS-CoV-2 infection, as well as the severity of COVID-19 based on pre-pandemic imaging alone. Does this mean that from the 1,955 participants mentioned in the result section, data of those wo died

and of those who refused to participate in the second imaging study, were analyzed for cross-sectional relationships?

Data from individuals from only one imaging time point at baseline was used only in the severity analysis (Tables 3 and 4)

7. Table 1: For smoking, alcohol consumption and selected diseases reported, the authors indicate that the percentage of the subjects is reported. However, from the data of that table the number cannot be percentages.

We have re-made the table to give percentages rather than fractions for categorical variables for easier reading.

8. Table 1: If the percentage of subjects having NAFLD amounts to 70-75, why was the percentage of patients with type 2 diabetes so low (2-3 percent). Considering the prevalence of NAFLD in patients with type 2 diabetes (Lancet Diabetes Endocrinol. 2022 Apr;10(4):284-296), these numbers should be different.

Thanks to the reviewer. We identified a bug in ascertainment of NAFLD cases, which we have now corrected. In fact, there is only a single participant with NAFLD diagnosed via tertiary care records (defined via ICD10 codes K74.0, K74.1, K74.2, K74.6, K76.0). We have therefore removed this from our analysis as we are not powered to detect differences in incidence or disease severity. To address the poor ascertainment of diabetes from tertiary care records, we have updated our analysis to use an expanded definition of diabetes (UK Biobank Fields 130708 and 130706 for T2D and T1D respectively) which incorporates primary care and self-report as well as tertiary care records. This affects table 1, table 3. We have removed supplementary table 2 as it was the same as T3.

9. Unexpectedly, the authors only observed a significant decrease in lung volume, but no change in any other examined image-derived phenotypes. May it be possible that most of the participants, who attended the second imaging study, had relatively mild COVID-19 and patients with a more severe course of the disease refused to participate in the second exam?

The referee raises an interesting point, and we too had expected to observe more significant changes in other organs given some reports in the literature, although many of these reports are from subjects with very severe disease. The majority of the UKBB subjects invited for a second scan and who had tested positive for COVID-19, had relatively mild disease (96% mild vs 4% severe). We do not have any information whether subjects with more severe disease were less likely to attend for follow-up scanning, but were reassured that the response rates in the COVID and control groups for repeat UKBB scanning were very similar, with a 58% response rate from participants who had COVID-19 and a 57% response rate from participants who have not been infected. It is however a valid point that there may have been some self-filtering where participants who had very severe COVID-19 (or

long-COVID effects) did not want to undergo a second scanning exam; we have added this as a caveat to the study.
